# Exploring Working Memory Capacity and Efficiency Processes to Understand Working Memory Training Outcomes in Primary School Children

**DOI:** 10.5334/joc.348

**Published:** 2024-02-08

**Authors:** Alexandra S. L. Tan, Regine C. Lau, Peter J. Anderson, Susan Gathercole, Mark A. Bellgrove, Joshua F. Wiley, Megan M. Spencer-Smith

**Affiliations:** 1 School of Psychological Sciences and Turner Institute for Brain and Mental Health, Monash University, Melbourne, Australia; 2 MRC Cognition and Brain Sciences Unit, Cambridge University, Cambridge, United Kingdom; 3 Department of Psychiatry, University of Cambridge, Cambridge, United Kingdom

**Keywords:** working memory training, cognitive training, training effects, transfer effects, cognitive outcomes

## Abstract

Despite the abundance of research evaluating working memory training outcomes in children, few studies have examined the underlying cognitive mechanisms. This study aimed to contribute understanding by exploring whether *working memory capacity* (maximum span) and/or *efficiency* (basic and cognitive processing speeds), two proposed cognitive mechanisms, are associated with children’s working memory performance immediately and 6-months post-intervention. We used data from a previous trial in primary school children (7–11 years) who completed working memory training (n = 52) or an active control (n = 36), comprising 10 sessions (each 20-minutes) in class over two weeks. Children completed five working memory measures at baseline, immediately and 6-months post-intervention: two Backwards Span and two Following Instructions measures (same paradigms as training activities), and one n-back measure (different paradigm). Maximum span, basic and cognitive processing speeds, and performance were calculated for each measure. Associations between change in maximum span, processing speeds and change in performance on the working memory measures from baseline to immediately and 6-months post-intervention did not differ between groups (all *p* < .05). Maximum span, processing speeds and performance on working memory measures did not differ between groups. Findings provide little evidence that the studied *capacity* or *efficiency* processes contribute to understanding working memory training outcomes in primary school children. Furthermore, working memory training did not have benefits for children’s working capacity, efficiency or performance up to 6-months post-intervention. It is of interest for future studies to explore cognitive mechanisms, including strategy use, maximum span and information processing, in datasets where training effects are observed.

## Introduction

There is evidence that working memory training can improve children’s performance on working memory measures (known as near transfer), at least in the short term (Gobet & Sala, 2020). Despite the abundance of research evaluating working memory training outcomes, few studies have examined the cognitive mechanisms underpinning training-induced improvements. Such knowledge would help guide families and practitioners in selecting interventions to support child development and inform the development of new interventions to achieve this potential.

Initially, researchers proposed that placing increasing demands on the working memory system by practicing activities with the difficulty adjusted to the trainee’s performance, the system would adapt to fundamentally improve working memory performance (Dahlin et al., 2008; Klingberg, 2010; Simons et al., 2016). The observed improvements following training were assumed to reflect increased *working memory capacity*, or the number of items that can be worked with in mind (Cowan, 2010). However, *working memory capacity* has been traditionally viewed as a stable trait that increases across childhood (Conway et al., 2005; Gathercole et al., 2004; Klingberg, 2010), and there has been little direct evidence of increased *capacity* resulting from training (Ericsson et al., 1980; Holmes et al., 2019; von Bastian et al., 2022).

There is increasing discussion based largely on adult work that working memory training involves learning new skills unique to the training activity (i.e. strategies), thus improving *cognitive efficiency* and in turn, performance on the activity and other similarly structured activities (near transfer measures) (Forsberg et al., 2020; Gathercole et al., 2019; Laine et al., 2018; von Bastian & Oberauer, 2014). Learning a new skill and completing controlled, concentrated processes also become largely automated with practice (e.g., Chein & Schneider, 2012, triarchic theory of learning), which can be reflected in faster processing speeds, including processing information with and without a cognitive load (cognitive and basic processing speeds) (Chiaravalloti et al., 2003; Gerst et al., 2021). Similarly, when learning strategies to complete a training activity (Gathercole et al., 2019), the information processing time decreases with continued practice (Heitz, 2014; Motes et al., 2018). Indeed, following strategy training alongside short-term memory training, adults showed faster response times and improved performance on working memory measures (McNamara & Scott, 2001). It is of interest to explore children’s processing speed following working memory training, given it could provide an estimate of improvements in cognitive efficiency including strategy, which changes across childhood and adulthood (Camos & Barrouillet, 2011; Chevalère et al., 2020).

Recent discussion acknowledges the potential contribution of multiple processes underpinning cognitive training outcomes (e.g., von Bastian et al., 2022, 2023; Zhang & Sauce, 2023). The *capacity-efficiency* model, for example, proposes that transfer reflects increased *cognitive capacity* (all available cognitive resources) and/or improved *cognitive efficiency* (optimised use of existing cognitive resources) (von Bastian et al., 2022). To date, no study has explored the contribution of proposed *capacity* or *efficiency* processes in children’s working memory training outcomes.

This exploratory study used data from a previous randomised controlled trial (RCT) evaluating working memory training methods in primary school children (Lau et al., 2023; submitted), and aimed to contribute understanding of cognitive mechanisms proposed in the capacity-efficiency model to underpin training outcomes by testing whether *working memory capacity* (maximum span) and/or *efficiency* (basic and cognitive processing speeds) are associated with children’s working memory performance (near transfer) immediately and 6-months post-intervention.

## Methods

### Design and participants

This study used data from a previous RCT evaluating methods of setting difficulty of working memory training activities in children (ACTRN12621000990820) (Lau et al., 2023; submitted). Children in Grades 2 to 5 (N = 201, 7–11 years) were recruited from one primary school in Melbourne, Australia and tested at baseline, immediately and 6-months post-intervention. Exclusion criteria were: caregiver-reported diagnosis of an intellectual disability and/or impairment in vision, hearing and/or fine motor skills that could not be corrected by aids, which would hinder their participation in the intervention and cognitive testing. Following baseline testing, children were allocated to working memory training (difficulty adaptive, self-select, stepwise) or active control conditions, stratified by age (7–8, 9–10, 11–12 years). Due to a technical error, an additional nine children 9–10 years and two children 11–12 years were allocated to the adaptive condition. Researchers, children, caregivers and teachers were blinded to group allocation and previous test results. Participants were blinded to study goals. The trial was approved by Monash University Human Research Ethics Committee (24305) and Melbourne Archdiocese Catholic Schools (1066). Caregivers provided informed consent and children provided assent.

The current study included children allocated to adaptive working memory training (n = 52) and active control (n = 36) conditions who completed all 10 sessions, and used their scores on working memory measures at baseline, immediately and 6-months post-intervention. Figure 1 in Supplementary material presents a flowchart of the previous trial highlighting the groups and data used in the current study.

### Intervention

Children participated in an experimental intervention developed in Minecraft: Education Edition (Lau et al., 2023), delivered by teachers in class on iPads with paediatric headphones to reduce distraction. It comprised 10 sessions, each 20-minutes, played over two weeks. Motivating features included the storyline (being an astronaut, discovering planets) and collecting experience points to use when exploring the world.

#### Adaptive working memory training

**Figure 1:**
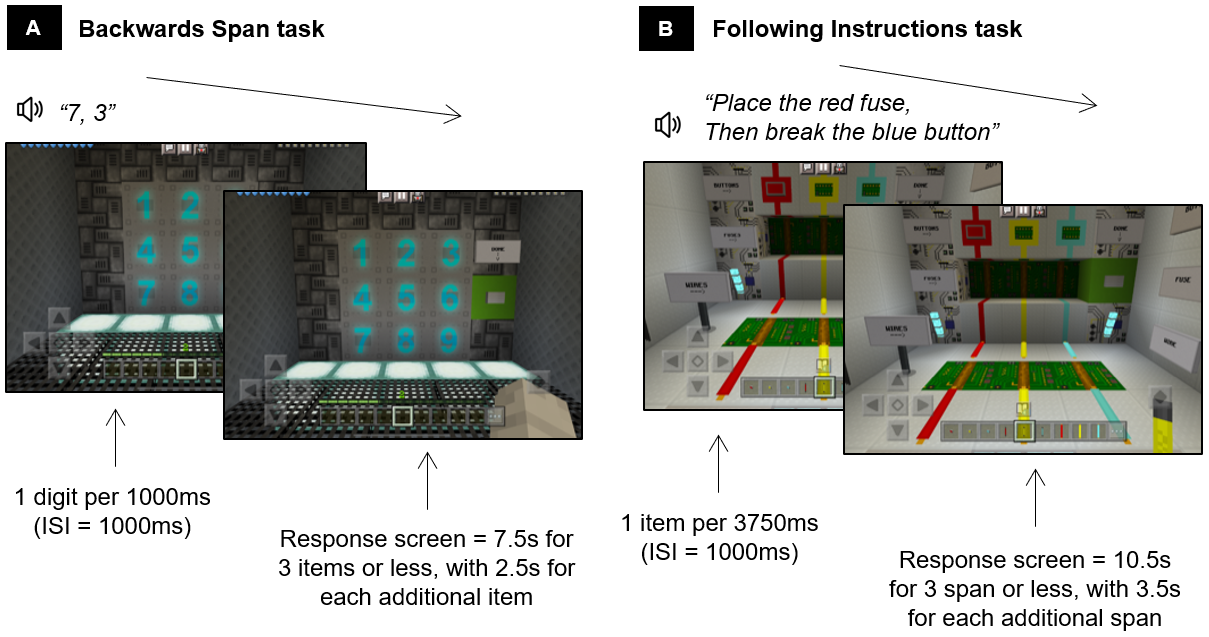
Adaptive working memory training activities: Backwards Span Digits activity **(A)** and Following Instructions Objects activity **(B)** modified from Lau et al. (2023). Note, ISI = interstimulus interval.

Children practiced two working memory activities each session that required temporarily storing and manipulating verbally presented information. A backward span activity (Figure 1A), based on the Digit Backward subtest from the WISC-V (Wechsler, 2014), required children to listen to a string of digits from one to nine and recall them in backwards order by tapping numbered buttons. A following instructions activity (Figure 1B), based on the task by Gathercole et al. (2008), where the child was introduced to three objects each coming in three colours, and two actions each requiring different tapping responses on the screen. The child had to remember and immediately complete an action-colour-object sequence(s), e.g., break the red wire. For each activity, the span level increased by one if three of four trials in a block (five blocks per session) were answered correctly and decreased by one if two consecutive trials in a block were answered incorrectly.

#### Active control

Each session children participated in new creative-building activities (not designed to train working memory), including building rooms in the rocket ship or structures on planets they visited. These activities took place within the same environment as the working memory training and thus, blinding to group allocation was maintained.

### Materials

**Figure 2:**
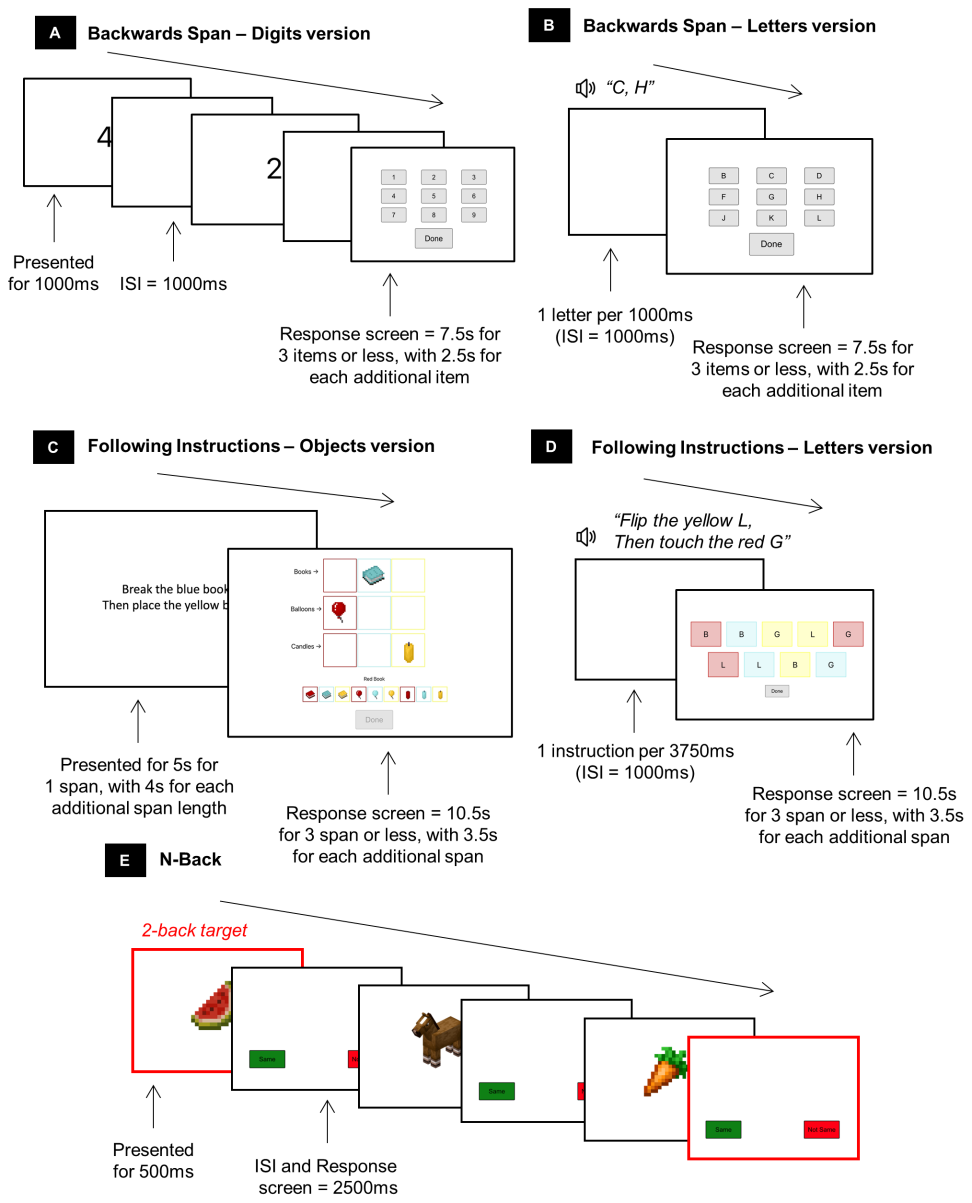
Working memory measures used to calculate performance, maximum span and processing speed: Backwards Span – Digits version **(A)** and Letters version **(B)**, Following Instructions – Objects version **(C)** and Letters version **(D)**, and N-back **(E)**, modified from Lau et al. (2023). Note. ISI = inter stimulus interval.

Children completed five working memory measures (Figure 2) at baseline, immediately and 6-months post-intervention on an iPad with paediatric headphones to reduce distraction (Lau et al., 2023). Testing was conducted at school in groups of 6–8 children in a quiet room by trained researchers during school hours. For each measure, maximum span, basic and cognitive processing speeds, and performance were calculated. Table 1 in Supplementary material presents reliability indices for each measure.

#### Working memory measures

##### Backwards Span – Digits and Letters versions

Children were required to recall a string of items in the reverse order of what was shown (digits version) or read aloud (letters version) by tapping buttons (Figure 2A and 2B). The span level began at two and increased by one if the child answered three of four trials in a block correctly and stopped if two or fewer trials in a block were correct.

##### Following Instructions – Objects and Letters versions

Children were required to place/break objects (objects version) or touch/flip letters (letters version) that differed by colour (Figure 2C and 2D). The span level began at one and increased by one if the child answered three of four trials in a block correctly and stopped if two or fewer trials in a block were correct.

##### N-back

This measure was designed based on the task by Katz et al. (2014). The child was shown in succession 22 common objects (6 targets, 16 non-targets), and had to tap the “same” button when the object shown was the same to that shown two objects previously or “not same” button when it was different (Figure 2E).

#### Capacity

Maximum span estimated *capacity*. For Backward Span and Following Instructions measures only, maximum span reflected the highest span a child reached where they correctly answered at least one of four trials in a block (e.g., Grogan et al., 2018; Holmes et al., 2019).

#### Efficiency

Two levels of processing speed, basic and cognitive (Salthouse, 2000), estimated *efficiency*. For each working memory measure, response time in milliseconds from the last stimulus presentation to when the child pressed the final response button for each included correct trial was recorded. Response times for the correct trials were averaged across the included trials and divided by the span level, providing an average response time per correct item. Basic processing speed was calculated for Backward Span and Following Instructions measures only from the block of four trials for the lowest span level, i.e., span of two on Backward Span measures, span of one on Following Instructions measures. Cognitive processing speed was calculated for Backward Span and Following Instructions measures from the block of four trials for the highest span level reached for a child, and for the N-back measure using all trials.

#### Performance

For Backwards Span and Following Instructions measures, performance reflected the total number of correct trials (maximum 32 and 36, respectively). For the N-back measure, performance reflected the total number of hits and correct rejections (maximum 20, given children are shown two objects before being able to respond).

### Data analysis

Data were analysed using R, version 4.2.1 (R Core Team, 2018). A series of regression models tested the contribution of change in the *capacity* (maximum span) or *efficiency* (basic or cognitive processing speed) score from baseline to immediately and 6-months post-intervention to change in the performance score from baseline to immediately and 6-months post-intervention on a working memory measure. In each model, the independent variable was an interaction term between change in the *capacity* or *efficiency* score (mean-centred) and group (training, control), and the dependent variable was change in the performance score on a working memory measure immediately or 6-months post-intervention. In analyses of interest, a series of regression models tested group differences in *capacity, efficiency* and performance on working memory measures immediately or 6-months post-intervention. In each model, the independent variable was group, and the dependent variable was *capacity, efficiency* or performance score on a working memory measure immediately or 6-months post-intervention. Covariates in all models were baseline score on the working memory measure and age (as used in stratification). Multiple comparisons were acknowledged by applying Bonferroni corrections. Sensitivity analyses adjusted for attention (ADHD Rating Scale-5 Home Version, ADHD-RS-5; DuPaul et al., 2016) or fluid intelligence (Raven’s Standard Progressive Matrices, SPM; Raven 1938, 1941). Interpretation of results considered effect sizes (Cohen’s *f*^2^; small = 0.02, medium = 0.15, large = 0.35; Cohen, 1988) and p-values.

## Results

Child characteristics (Table 1) and working memory scores (Table 2) were largely similar for the training and control groups at baseline, although the training group was older, had slower cognitive processing speed on Backwards Span Digits, and higher maximum span and faster cognitive processing speed on Following Instructions Objects.

**Table 1:** Child Characteristics at Baseline.


	WORKING MEMORY TRAINING GROUP (n = 52)	ACTIVE CONTROL GROUP (n = 36)	GROUP DIFFERENCE *p*

Age in years, mean (SD)	9.43 (1.31)	8.84 (1.07)	0.02

Female sex, n (%)	23 (44.20)	16 (44.44)	0.95

Primary caregiver highest level of education, n (%)			0.70

Completed high school	2 (3.85)	1 (2.78)	

University bachelor degree/diploma	23 (44.23)	16 (44.44)	

University postgraduate degree	6 (11.54)	7 (19.44)	

Not reported	21 (40.39)	12 (33.33)	

Secondary caregiver highest level of education, n (%)			0.50

Did not complete high school	0 (0.00)	1 (2.78)	

Completed high school	4 (6.35)	1 (2.78)	

University bachelor degree/diploma	19 (30.16)	14 (38.89)	

University postgraduate degree	12 (19.05)	6 (16.67)	

Not reported	28 (44.44)	14 (38.89)	

ADHD-RS-5 (raw score), mean (SD)^1^	9.25 (9.06)	12.16 (10.96)	0.36

Raven’s SPM (raw score), mean (SD)^2^	34.77 (9.11)	30.53 (9.77)	0.60


*Note*: ^1^One child in the control had ratings of clinical concern for ADHD-RS-5 and ^2^one child in each group had scores of clinical concern for the Raven’s SPM. Regressions and Chi-square tests were used to test group differences at baseline. Analyses adjusted for baseline age when testing group differences in scores for ADHD-RS-5 and Raven’s SPM. Abbreviations: ADHD-RS-5, ADHD Rating Scale-5 Home Version (18 items of inattention and hyperactivity/impulsivity symptoms rated on a 4-point scale by caregivers of children 5–17 years yield a total score ranging 0–54 with higher scores indicate greater symptoms. Scores >93^rd^ percentile indicate clinical concern). Raven’s SPM, Raven’s Standard Progressive Matrices (a measure of fluid intelligence for individuals 6.5–80 years comprising five sets of 12 puzzles where the individual must identify the missing piece with the total score ranging 0–60 with higher scores indicating better fluid intelligence. Scores <7^th^ percentile indicate clinical concern).

**Table 2:** Working Memory Scores at Baseline, Immediately Post-Intervention and 6-months Post-Intervention.


	WORKING MEMORY TRAINING GROUP	ACTIVE CONTROL GROUP	*p*	*f* ^2^	WORKING MEMORY TRAINING GROUP	ACTIVE CONTROL GROUP	*p*	*f* ^2^	WORKING MEMORY TRAINING GROUP	ACTIVE CONTROL GROUP	*p*	*f* ^2^

BACKWARDS SPAN DIGITS, MEAN (SD)												

	BASELINE				IMMEDIATELY POST-INTERVENTION				6-MONTHS POST-INTERVENTION			

Performance	7.60 (3.08)	6.62 (3.19)	0.48	0.01	7.59 (4.20)	6.51 (3.80)	0.94	0.00	9.00 (4.63)	8.03 (3.38)	0.88	0.00

Maximum Span	3.56 (0.85)	3.24 (1.02)	0.26	0.01	3.53 (1.14)	3.20 (1.11)	0.80	0.00	4.00 (1.44)	3.60 (1.06)	0.56	0.00

Basic Processing Speed	1396.20 (368.87)	1492.85 (618.72)	0.98	0.00	1468.11 (479.21)	1643.09 (473.68)	0.50	0.01	1219.15 (323.48)	1348.80 (331.11)	0.52	0.01

Cognitive Processing Speed	1672.74 (488.10)	1450.39 (497.74)	0.02	0.08	1906.80 (1534.72)	1698.71 (886.39)	0.71	0.00	1425.57 (439.33)	1398.37 (342.74)	0.49	0.01

**BACKWARDS SPAN LETTERS, MEAN (SD)**												

	**BASELINE**				**IMMEDIATELY POST-INTERVENTION**				**6-MONTHS POST-INTERVENTION**			

Performance	5.50 (2.44)	5.00 (2.19)	0.86	0.00	6.39 (3.30)	5.66 (2.42)	0.90	0.00	6.78 (2.83)	5.91 (2.36)	0.39	0.01

Maximum Span	2.88 (0.81)	2.72 (0.88)	0.91	0.00	3.16 (1.12)	2.97 (0.79)	0.60	0.00	3.34 (0.82)	3.06 (0.73)	0.31	0.01

Basic Processing Speed	1933.59 (634.04)	1894.79 (515.96)	0.25	0.02	1657.46 (499.58)	1968.76 (614.34)	0.12	0.03	1709.85 (561.23)	1827.99 (472.22)	0.92	0.00

Cognitive Processing Speed	1871.48 (553.58)	1872.35 (654.26)	0.94	0.00	1812.67 (385.60)	1833.66 (504.96)	0.94	0.00	1703.80 (472.43)	1594.43 (440.50)	0.12	0.04

**FOLLOWING INSTRUCTIONS OBJECTS, MEAN (SD)**												

	**BASELINE**				**IMMEDIATELY POST-INTERVENTION**				**6-MONTHS POST-INTERVENTION**			

Performance	7.12 (2.19)	5.72 (2.31)	0.05	0.05	7.06 (2.42)	5.80(2.32)	0.15	0.03	7.44 (3.06)	6.69 (2.45)	0.75	0.00

Maximum Span	2.33 (0.62)	1.89 (0.58)	0.01	0.08	2.37 (0.76)	1.94 (0.68)	0.09	0.04	2.48 (0.86)	2.26 (0.70)	0.69	0.00

Basic Processing Speed	4820.88 (1445.40)	5269.37 (1759.51)	0.63	0.00	3478.83 (924.57)	4222.04 (1422.01)	0.03	0.06	3443.72 (984.84)	3766.26 (1092.09)	0.64	0.00

Cognitive Processing Speed	3199.25 (933.99)	4240.08 (1968.37)	0.01	0.12	2713.86 (860.50)	3044.82 (939.46)	0.43	0.01	2696.12 (698.30)	3045.40 (1265.15)	0.25	0.02

**FOLLOWING INSTRUCTIONS LETTERS, MEAN (SD)**												

	**BASELINE**				**IMMEDIATELY POST-INTERVENTION**				**6-MONTHS POST-INTERVENTION**			

Performance	6.56 (1.41)	5.66 (2.04)	0.09	0.03	6.21 (2.13)	5.71 (2.22)	0.68	0.00	6.06 (2.08)	5.66 (1.75)	0.59	0.00

Maximum Span	2.00 (0.34)	1.83 (0.57)	0.31	0.01	2.25 (0.64)	1.86 (0.49)	0.01	0.08	2.00 (0.61)	1.97 (0.57)	0.89	0.00

Basic Processing Speed	3225.83 (1552.82)	4111.80 (2748.91)	0.20	0.02	3251.00 (1515.78)	3598.16 (1623.54)	0.79	0.00	2491.87 (845.85)	2855.11 (984.83)	0.37	0.01

Cognitive Processing Speed	2662.68 (763.73)	3636.20 (2199.47)	0.09	0.08	2809.44 (1480.79)	3023.95 (1353.23)	0.87	0.00	2369.98 (755.84)	2228.71 (513.51)	0.25	0.03

**N-BACK, MEAN (SD)**												

	**BASELINE**				**IMMEDIATELY POST-INTERVENTION**				**6-MONTHS POST-INTERVENTION**			

Performance	13.19 (5.04)	10.06 (5.55)	0.06	0.04	14.80 (4.11)	11.50 (5.38)	0.02	0.07	16.10 (3.59)	14.00 (4.34)	0.08	0.04

Cognitive Processing Speed	1332.78 (329.55)	1425.74 (417.39)	0.23	0.02	1330.68 (321.82)	1408.47 (374.46)	0.46	0.01	1223.22 (283.39)	1341.29 (360.36)	0.28	0.01


*Note*: *p* Group difference p-value, *f*^2^ Cohen’s effect size (small = 0.02, medium = 0.15, large = 0.35). Regressions and Chi-square tests were used to test group differences in working memory measures. Analyses adjusted for baseline age when testing group differences in scores.

### Associations between changes in capacity or efficiency and changes in performance on working memory measures in the context of working memory training compared with the control

There were no interactions between group and change scores from baseline to immediate or 6-months post-intervention observed across the working memory measures (Table 3). Patterns of small effect sizes were noted, with interactions between group and change score being in the expected direction: 1) immediately post-intervention for three of five cognitive processing speed scores, and 2) 6-months post-intervention for two of four maximum span scores, two of four basic processing speed scores, and two of five cognitive processing speed scores.

**Table 3:** Moderated Multiple Regression Results for Change in Working Memory Performance From Baseline to Immediately and 6-months Post-intervention Predicted by Change in Maximum Span, Basic Processing Speed or Cognitive Processing Speed, and an Interaction Between the Change Score and Group.


OUTCOME MEASURE/PREDICTOR	*B* [95% CONFIDENCE INTERVALS]	β	*SE*	*f* ^2^	*B* [95% CONFIDENCE INTERVALS]	β	*SE*	*f* ^2^

MAXIMUM SPAN								

	IMMEDIATELY POST-INTERVENTION				6-MONTHS POST INTERVENTION			

**Backwards Span Digits**								

Maximum span change	3.01*** [2.40, 3.63]	0.84	0.31	1.24	2.34*** [1.85, 2.83]	0.73	0.25	1.14

Change * Group	0.06 [–0.71, 0.82]	0.01	0.38	0.00	0.70* [0.13, 1.28]	*0.18*	*0.29*	*0.07*

**Backwards Span Letters**								

Maximum span change	1.91*** [1.38, 2.43]	0.72	0.26	0.67	1.87*** [1.14, 2.60]	0.64	0.37	0.33

Change * Group	0.36 [–0.24, 0.96]	0.11	0.30	0.02	0.57 [–0.27, 1.41]	0.16	0.42	0.02

**Following Instructions Objects**								

Maximum span change	2.46*** [1.87, 3.05]	0.78	0.30	0.88	2.71*** [2.09, 3.33]	0.75	0.31	0.95

Change * Group	–0.37 [–1.08, 0.34]	–0.09	0.36	0.01	0.35 [–0.44, 1.14]	0.07	0.40	0.01

**Following Instructions Letters**								

Maximum span change	2.78*** [2.13, 3.43]	0.81	0.33	0.95	2.12*** [1.50, 2.74]	0.62	0.31	0.59

Change * Group	–0.27 [–1.11, 0.58]	–0.06	0.42	0.01	0.36 [–0.56, 1.27]	0.07	0.46	0.01

**BASIC PROCESSING SPEED**								

	**IMMEDIATELY POST-INTERVENTION**				**6-MONTHS POST INTERVENTION**			

**Backwards Span Digits**								

Basic processing speed change	<–0.001 [–0.002, <0.001]	–0.13	0.00	0.01	<0.001 [–0.001, 0.002]	0.08	0.00	0.01

Change * Group	<–0.001 [–0.002, 0.002]	–0.01	0.00	0.00	–0.002 [–0.006, <0.001]	–0.19	0.00	0.03

**Backwards Span Letters**								

Basic processing speed change	<–0.001 [–0.002, <0.001]	–0.16	0.00	0.01	<–0.001 [–0.002, <0.001]	–0.14	0.00	0.01

Change * Group	<0.001 [–0.001, 0.002]	0.10	0.00	0.00	<0.001 [–0.001, 0.001]	0.04	0.00	0.00

**Following Instructions Objects**								

Basic processing speed change	<–0.001 [<–0.001, <0.001]	–0.03	0.00	0.00	<–0.001 [<0.001, <0.001]	–0.21	0.00	0.03

Change * Group	<0.001 [<–0.001, <0.001]	0.10	0.00	0.01	<0.001 [<0.001, <0.001]	0.08	0.00	0.00

**Following Instructions Letters**								

Basic processing speed change	<–0.001 [<–0.001, <0.001]	–0.01	0.00	0.00	<–0.001 [0.00, 0.00]	–0.05	0.00	0.00

Change * Group	<–0.001 [<–0.001, <0.001]	–0.14	0.00	0.02	<–0.001* [–0.001, <0.001]	*–0.26*	*0.00*	*0.07*

**COGNITIVE PROCESSING SPEED**								

	**IMMEDIATELY POST–INTERVENTION**				**6–MONTHS POST INTERVENTION**			

**Backwards Span Digits**								

Group (training group)	0.001* [<0.001, 0.003]	*0.68*	*0.00*	*0.12*	<0.001 [–0.004, 0.004]	0.00	0.00	0.00

Change * Group	–0.001 [<–0.001, <0.001]	–0.42	0.00	0.05	<0.001 [–0.004, 0.005]	0.06	0.00	0.00

**Backwards Span Letters**								

Cognitive processing speed change	<–0.001 [–0.002, 0.001]	–0.06	0.00	0.00	0.002* [<0.001, 0.003]	*0.48*	*0.00*	*0.13*

Change * Group	<0.001 [–0.002, 0.002]	0.09	0.00	0.00	–0.001 [–0.003, <0.001]	–0.38	0.00	0.09

**Following Instructions Objects**								

Cognitive processing speed change	<–0.001 [<–0.001, <0.001]	–0.12	0.00	0.01	<–0.001* [–0.001, <0.001]	*–0.35*	*0.00*	*0.19*

Change * Group	<–0.001 [<–0.001, <0.001]	–0.26	0.00	0.08	<–0.001 [–0.001, <0.001]	–0.05	0.00	0.00

**Following Instructions Letters**								

Cognitive processing speed change	<–0.001 [<–0.001, <0.001]	–0.24	0.00	0.04	<0.001 [<0.001, <0.001]	0.10	0.00	0.01

Change * Group	<–0.001 [<–0.001, <0.001]	–0.15	0.00	0.02	<–0.001 [–0.002, <0.001]	–0.33	0.00	0.13

**N-Back**								

Cognitive processing speed change	–0.001 [–0.003, <0.001]	–0.16	0.00	0.01	–0.001 [–0.003, <0.001]	–0.20	0.00	0.02

Change * Group	0.001 [–0.002, 0.004]	0.11	0.00	0.01	<0.001 [–0.002, 0.003]	0.07	0.00	0.00


*Note*: *B* unstandardised beta (negative value for an interaction indicates the training group had a lower score), SE standard error of the estimate, β standardised beta, *f*^2^ Cohen’s effect size (small = 0.02, medium = 0.15, large = 0.35), * p < 0.05, ** p < 0.01, *** p < 0.005. To apply Bonferroni corrections, significance levels of *p* = .004 (adjusting for 13 tests at each time-point) were calculated. Italics indicate non-significance after the Bonferroni correction was applied.

### Group differences in capacity, efficiency and performance

Maximum span, processing speeds and performance on working memory measures immediately and 6-months post-intervention did not differ statistically between the training and control groups (Table 4). Patterns of small effect sizes were observed, with the training group performing better than the control: 1) immediately post-intervention for two of four maximum span scores, two of four basic processing speed scores and two of five cognitive processing speed scores, and 2) 6-months post-intervention for three of five cognitive processing speed scores.

**Table 4:** Multiple Regression Results for Working Memory Performance, Capacity (Maximum Span) and Efficiency (Basic and Cognitive Processing Speeds) Scores Immediately and 6-months Post-intervention Predicted by Group, Covarying for Baseline Score and Age.


OUTCOME MEASURE/PREDICTOR	*B* [95% CONFIDENCE INTERVALS]	β	*SE*	*f* ^2^	*B* [95% CONFIDENCE INTERVALS]	β	*SE*	*f* ^2^

PERFORMANCE								

	IMMEDIATELY POST-INTERVENTION				6-MONTHS POST INTERVENTION			

**Backwards Span Digits**								

Group (training group)	–0.18 [–1.44, 1.08]	–0.02	0.63	0.00	–0.02 [–1.67, 1.63]	0.00	0.83	0.00

**Backwards Span Letters**								

Group (training group)	–0.11 [–1.13, 0.91]	–0.02	0.51	0.00	0.51 [–0.52, 1.55]	0.09	0.52	0.01

**Following Instructions Objects**								

Group (training group)	0.42 [–0.48, 1.31]	0.08	0.45	0.01	0.02 [–1.18, 1.23]	0.00	0.61	0.00

**Following Instructions Letters**								

Group (training group)	0.01 [–0.94, 0.96]	0.00	0.48	0.00	0.13 [–0.73, 1.00]	0.03	0.43	0.00

**N-Back**								

Group (training group)	1.64 [–0.03, 3.32]	0.17	0.84	0.05	1.14 [–0.55, 2.83]	0.14	0.85	0.02

**MAXIMUM SPAN**								

	**IMMEDIATELY POST-INTERVENTION**				**6-MONTHS POST-INTERVENTION**			

**Backwards Span Digits**								

Group (training group)	–0.06 [–0.42, 0.30]	–0.03	0.64	0.00	0.08 [–0.44, 0.61]	0.03	0.26	0.00

**Backwards Span Letters**								

Group (training group)	–0.10 [–0.45, 0.25]	–0.05	0.63	0.00	0.19 [–0.13, 0.50]	0.12	0.16	0.02

**Following Instructions Objects**								

Group (training group)	0.19 [–0.10, 0.47]	0.12	0.49	0.02	–0.01 [–0.36, 0.34]	–0.01	0.17	0.00

**Following Instructions Letters**								

Group (training group)	0.30* [ 0.04, 0.56]	*0.25*	*0.47*	*0.07*	–0.01 [–0.27, 0.25]	–0.01	0.13	0.00

**BASIC PROCESSING SPEED**								

	**IMMEDIATELY POST-INTERVENTION**				**6-MONTHS POST-INTERVENTION**			

**Backwards Span Digits**								

Group (training group)	–65.08 [–252.30, 122.13]	–0.07	452.62	0.01	–41.83 [–171.06, 87.39]	–0.06	64.92	0.01

**Backwards Span Letters**								

Group (training group)	–226.04* [–434.11, –17.96]	*–0.20*	*477.61*	*0.06*	9.70 [–195.00, 214.41]	0.01	102.84	0.00

**Following Instructions Objects**								

Group (training group)	–540.01* [–1039.64, –40.39]	*–0.22*	*250.96*	*0.06*	–88.06 [–512.35, 336.23]	–0.04	213.16	0.00

**Following Instructions Letters**								

Group (training group)	107.11 [–540.03, 754.24]	0.03	324.99	0.00	–99.75 [–461.82, 262.33]	–0.05	181.87	0.00

**COGNITIVE PROCESSING SPEED**								

	**IMMEDIATELY POST–INTERVENTION**				**6–MONTHS POST INTERVENTION**			

**Backwards Span Digits**								

Group (training group)	–91.20 [–1014.85, 832.46]	–0.03	458.59	0.00	–30.22 [–275.03, 214.59]	–0.04	121.76	0.00

**Backwards Span Letters**								

Group (training group)	55.94 [–252.27, 364.15]	0.06	151.97	0.00	218.41 [ –68.72, 505.55]	0.24	141.84	0.06

**Following Instructions Objects**								

Group (training group)	–285.76 [–955.32, 383.80]	–0.15	328.71	0.02	–415.22 [–1132.04, 301.59]	–0.19	354.67	0.03

**Following Instructions Letters**								

Group (training group)	901.38 [ –506.28, 2309.03]	0.26	683.48	0.07	237.42 [–217.56, 692.39]	0.21	219.94	0.05

**N-Back**								

Group (training group)	–41.20 [–191.82, 109.42]	–0.06	75.67	0.00	–59.47 [–192.70, 73.77]	–0.09	66.96	0.01


*Note*: *B* unstandardised beta (negative value for an interaction indicates the training group had a lower score), SE standard error of the estimate, β standardised beta, *f*^2^ Cohen’s effect size (small = 0.02, medium = 0.15, large = 0.35), * p < 0.05, ** p < 0.01, *** p < 0.005. To apply Bonferroni corrections, significance levels of *p* = .003 (adjusting for 18 tests at each time-point) were calculated. Italics indicate non-significance after the Bonferroni correction was applied.

**Figure 3:**
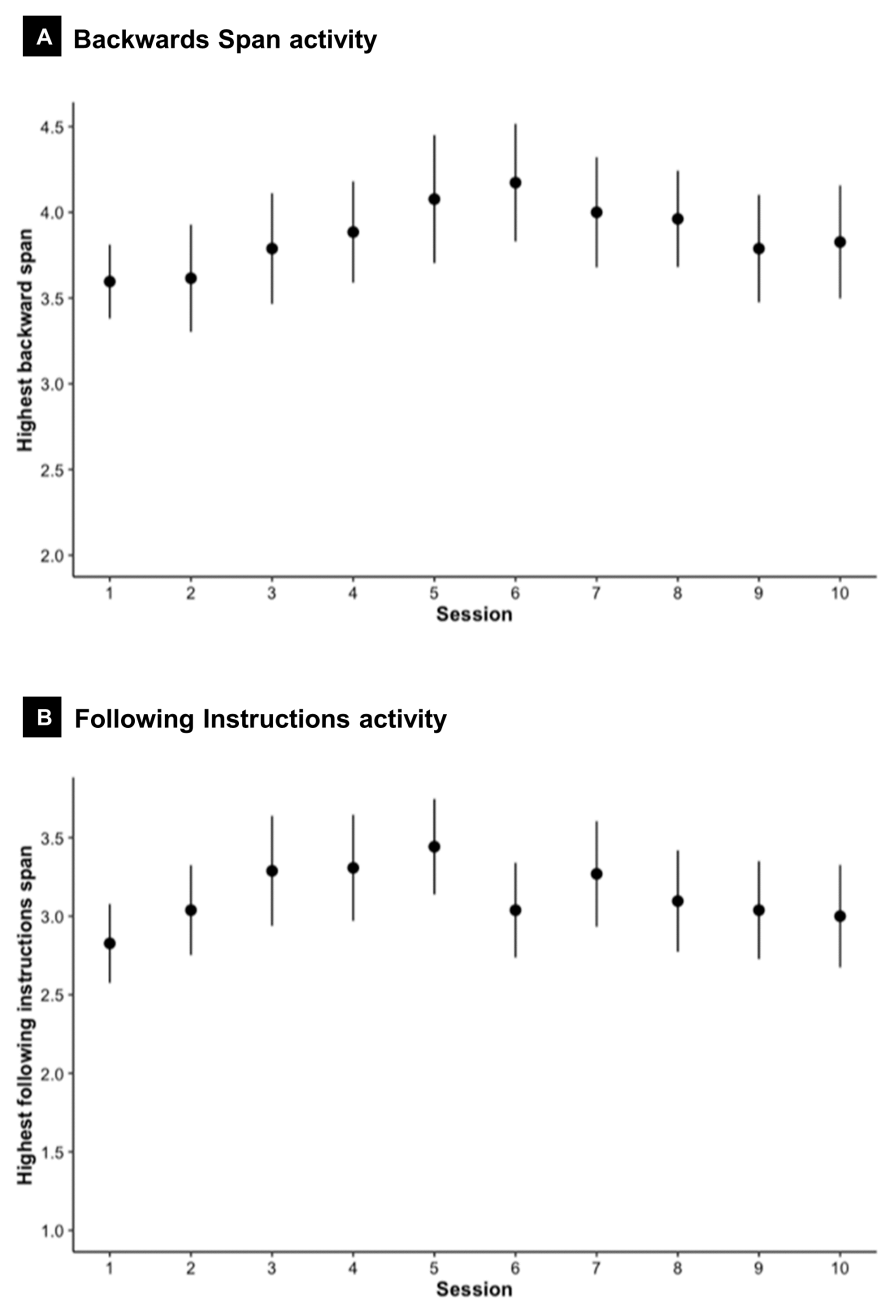
Mean highest span level trained on (and 95% CIs) for each training session in the adaptive working memory training group: Backwards Span Digits activity **(A)** and Following Instructions Objects activity **(B)**.

Of interest, children in the working memory training condition increased the span level trained on during the intervention period for both the Backward Span and Following Instructions activities (lowest span 2 and 1, respectively), Figure 3.

### Sensitivity analyses

Results were similar after covarying for ADHD-RS-5 or Raven’s SPM scores (results not shown).

## Discussion

We found little evidence that working memory *capacity* (maximum span) and/or *efficiency* (basic and cognitive processing speeds) processes contribute to understanding working memory training outcomes (near transfer) in primary school children immediately or 6-months post-intervention. These findings are in contrast to discussions in the literature that cognitive capacity and efficiency processes have an important role in the mechanisms underpinning working memory training outcomes (e.g., von Bastian et al., 2022, 2023; Zhang & Sauce, 2023), and might reflect the lack of training effects we observed for working memory outcomes.

Our results revealed no associations between changes in capacity or efficiency and changes in performance on a range of near transfer measures for primary school children in the context of working memory training compared with an active control. While we observed some patterns of small effect sizes, replication of results is needed to help determine whether they are meaningful (Carey et al., 2023). Although these are new findings, they appear inconsistent with development studies reporting important links between children’s capacity and efficiency and their performance on working memory measures (Bayliss et al., 2005; Gathercole et al., 2004), thought to reflect typical development where capacity (Gathercole et al., 2004; Simmering & Perone, 2013) and efficiency (Kail, 2007; Nettelbeck & Burns, 2010) improve with age alongside working memory performance (Gathercole et al., 2004; Simmering & Perone, 2013).

Analyses of interest revealed no benefits of working memory training for children’s capacity, efficiency or performance on working memory outcome measures compared with the active control. Our null effects might be unexpected given children improved on the training activities (Figure 3), and results are in contrast to conclusions in the training literature describing evidence of near transfer effects in children (Gobet & Sala, 2020). However, previous child studies frequently report improvements only on select (not all) working memory measures used. For example, the Memory Maestros study found that Grade 1 children in the working memory training group (n = 226) compared with usual teaching (n = 226) showed significantly better performance on two of the four working memory measures at 6-months post-randomisation (Roberts et al., 2016).

Our study methods are important to consider in drawing conclusions from the null outcome effects. We studied children attending primary school who would be expected to function at (near) optimal levels, with less room for improvement and thus unlikely to gain from training. This is consistent with reasoning and evidence in children in favour of the compensation perspective of understanding individual differences in training outcomes (e.g. Lövden et al., 2012), rather than the magnification perspective (e.g., Sala et al., 2019), of understanding individual differences in training outcomes. We note, children in our training group were older and tended to perform better on baseline measures, which may have contributed to the observed null effects. Our findings should not be generalised to other populations, such as children with low working memory. The active control comprised creative building activities, which may have unintentionally recruited working memory. The working memory training comprised a backward span activity shown to have large transfer (e.g., d = 0.90; Gathercole et al., 2019) and a following instructions activity not previously used for training, practice was intensive (many training studies and commercial programs use intensive training schedules; see Gathercole et al., 2019) and dose was relatively low at 200 min (compared with, for example, ~900 min in the Memory Maestro study; Roberts et al., 2016). Given limited evidence of optimal training paradigms, schedules or dose in children, it is not clear whether and/or how these design features influenced results. Children improved on the training activities, but it is not clear whether a certain amount of improvement is needed for transfer. The range of working memory measures, four having the same paradigms as the training activities (Backward Span, Following Instruction) and one a different paradigm (N-back), provides confidence in the results observed across all measures.

Strengths of this study include exploring two proposed cognitive mechanisms, instead of focusing on *capacity* or *efficiency* (e.g., Dunning & Holmes, 2014; McNamara & Scott, 2001). Two levels of information processing (basic and cognitive processing speed) were examined, contributing understanding of the unique contribution of this proposed mechanism to working memory training outcomes. Using five working memory measures provided confidence in our findings (Shipstead et al., 2010), with studies often using one (e.g., Dahlin et al., 2008). Employing an active control group ruled out possible placebo and/or expectation effects (Gobet & Sala, 2022; Simons et al., 2016; von Bastian et al., 2022). Our large sample size (n = 113) provided confidence in the study findings. This is among the few studies examining proposed cognitive mechanisms underpinning children’s working memory training outcomes (e.g., Ding et al., 2019; Gathercole et al., 2019).

Study limitations include the post-hoc design, which meant we could only use data collected as part of the previous study (Lau et al., 2023; submitted) to study potential mechanisms, which did not include strategy use. There is growing evidence in adults that strategy use is a key cognitive mechanism underpinning working memory training outcomes (Forsberg et al., 2020; Gathercole et al., 2019; Laine et al., 2018; von Bastian et al., 2022), and is associated with improvements in maximum span (McNamara & Scott, 2001) and information processing (Ding et al., 2019; Laine et al., 2018). Strategy use differs across childhood and adulthood (Camos & Barrouillet, 2011; Chevalère et al., 2020), thus exploring the role of children’s strategy use in understanding working memory training outcomes is of interest. Group differences in age and performance at baseline, although adjusted for in analyses, could have impacted results. Split-half reliability for our Following Instructions tasks was low. Our approach of using individual outcome measures in analyses rather than latent constructs could be considered a limitation. Our capacity and performance measures might be confounded, given the working memory measures (with the exception of the N-back) and training activities increased in difficulty following accurate performance and thus could be considered to emphasise accuracy. The maximum span score we used was conservative and may not have been indicative of a child’s highest possible span because of the design of the working memory measures (a child advanced a span level if they answered correctly three of four trials in a block).

We believe there is value for future research to study mechanisms underpinning training outcomes in datasets where training effects are observed, using performance on training activities and outcome measures. Examining multiple processes (e.g., strategy use, maximum span, information processing) to understand how they may work together or uniquely contribute to understanding children’s training outcomes is of interest. Measures should be designed to reduce potential confounds.

## Conclusion

Our findings provide little evidence that the examined *working memory capacity* (maximum span) or *efficiency* processes (basic and cognitive processing speed) are associated with near transfer in primary school children. We did not observe benefits of working memory training for children’s working memory capacity, efficiency or performance up to 6-months post-intervention. Of concern, families and practitioners still likely do not know how to determine whether a specific cognitive training intervention may benefit a child. We provide a starting point for future studies examining cognitive mechanisms underpinning children’s working memory training outcomes, knowledge important for informing the development of effective training interventions.

## Data Accessibility Statement

The research ethics approval prevents us from making de-identified data available in a public repository, but allows de-identified data to be shared for future ethically approved research. To request and receive data please contact the study investigators Megan.Spencer-Smith@monash.edu, Peter.J.Anderson@monash.edu and Joshua.Wiley@monash.edu.

## Additional File

The additional file for this article can be found as follows:

10.5334/joc.348.s1 Supplementary material. Figure 1 and Table 1.
