# Supplementary material for: Exploring Working Memory Capacity and Efficiency Processes to Understand Working Memory Training Outcomes in Primary School Children
Source: J Cogn. 2024 Feb 8;7(1):23. doi: 10.5334/joc.348 (PMC10877965; doi:10.5334/joc.348)
Supplement: Supplementary material. — Figure 1 and Table 1. [file joc-7-1-348-s1.pdf]

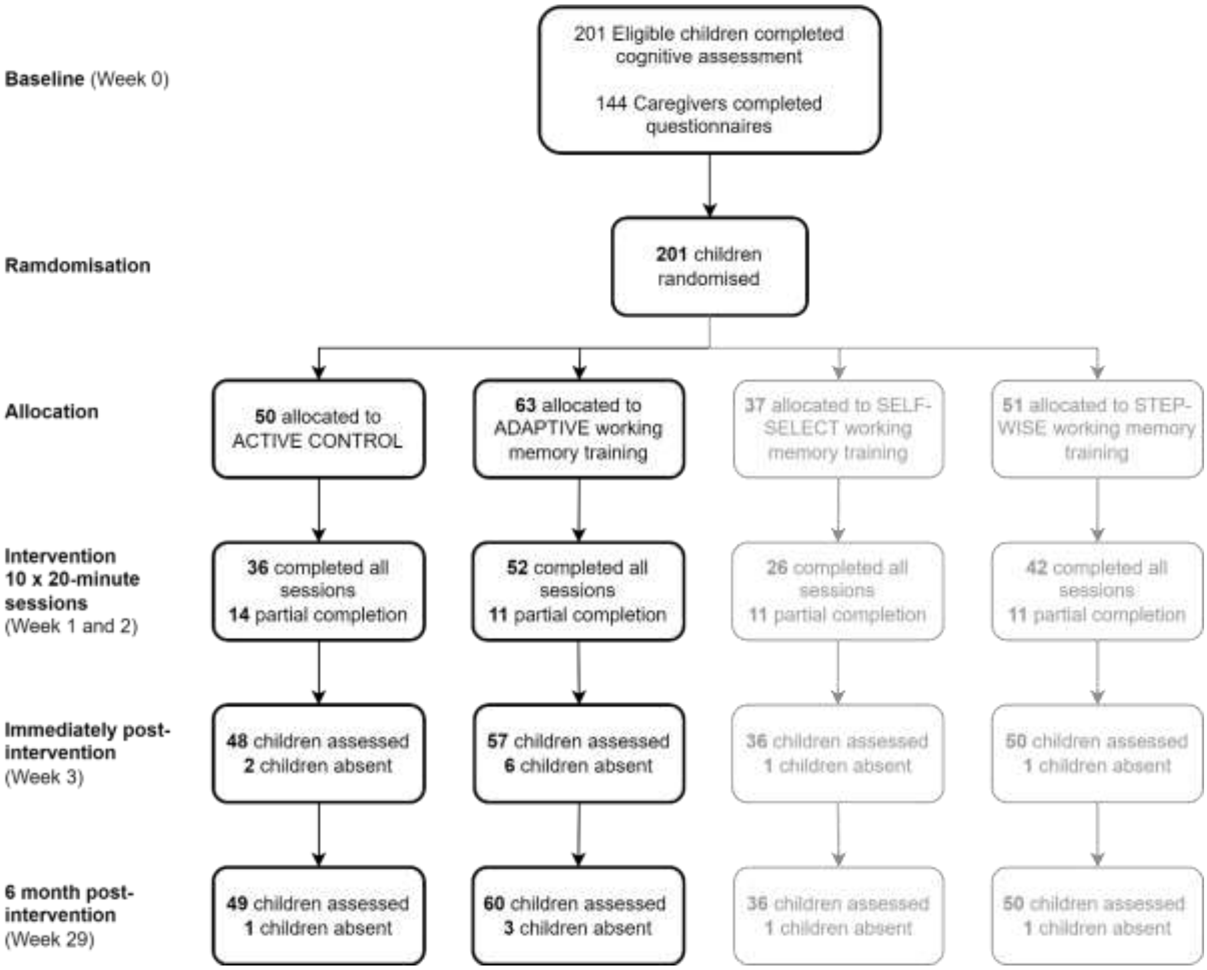

Figure 1. Participant flow, modified from Lau et al. (2023).

Note. There were more children in the adaptive working memory training condition due to a technical error by a team member not involved in data collection, which resulted in an additional 9 children aged 9-10 years and 2 children aged 11-12 years in the group.

Table 1.

Split-half reliability indices (odd-even method with Spearman-Brown correction) for working memory measures at each timepoint Lau et al. (submitted).

	**Split-half reliability indices**		
	Baseline	Immediately Post-Intervention	6-months Post-Intervention
Backwards Span Digits	0.84	0.88	0.86
Backwards Span Letters	0.81	0.86	0.82
Following Instructions Objects	0.65	0.55	0.71
Following Instructions Letters	0.32	0.59	0.61
N-Back	0.89	0.87	0.85
